# Persistent hypoglycemia due to an IGF‐II‐secreting malignant pheochromocytoma: a case report and literature review

**DOI:** 10.1002/ccr3.3161

**Published:** 2020-07-23

**Authors:** María Martínez García, Pablo Trincado Aznar, María Elena López Alaminos, Mikel González Fernández, Almendra Alvarado Rosas, Martín Laclaustra Gimeno

**Affiliations:** ^1^ Endocrinology Miguel Servet University Hospital Zaragoza Spain; ^2^ Translational Research Unit Miguel Servet University Hospital Zaragoza Spain

**Keywords:** case report, hypoglycemia, IGF‐II‐induced hypoglycemia, nonislet cell hypoglycemia, pheochromocytoma

## Abstract

A malignant pheochromocytoma with IGF‐II‐mediated hypoglycemia is reported; although treatment was cumbersome and evolution unfortunate, this diagnosis must be kept in mind when dealing with NICTH's differential diagnosis.

## Graphical abstract

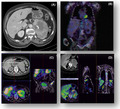

## 1 INTRODUCTION

Nonislet cell tumor hypoglycemia (NICTH) constitutes an underdiagnosed cause of hypoglycemia due to its rarity, ambiguous laboratory picture, and atypical clinical presentation. It is caused by an excessive production of aberrant IGF‐II or pro‐IGF‐II in association with multiple tumors (especially epithelial and mesenchymal)^1^ that potentially influences management strategy.^2^ In contrast, by excessive production of catecholamines, pheochromocytomas frequently cause hyperglycemia due to a combination of a pro‐inflammatory state and a frank deterioration of early phasic release of insulin, which seems to be the predominant mechanism.^3^ Nevertheless, in up to 13% of patients after‐surgery hypoglycemia due to impair glucagon secretion may occur, especially in epinephrine secreting tumors with phentolamine preoperative α‐blockade. Fatal hypoglycemia due to GLP‐1 production or extremely high tumor consumption of glucose, as evidenced by PET, has been exceptionally reported in malignant pheochromocytoma. Only two pheochromocytomas with insulin‐mediated hypoglycemia have been published so far, but no reports indicate IGF‐II operating in this particular scenario.

### 1.1 CASE SCENARIO

**FIGURE 1:**
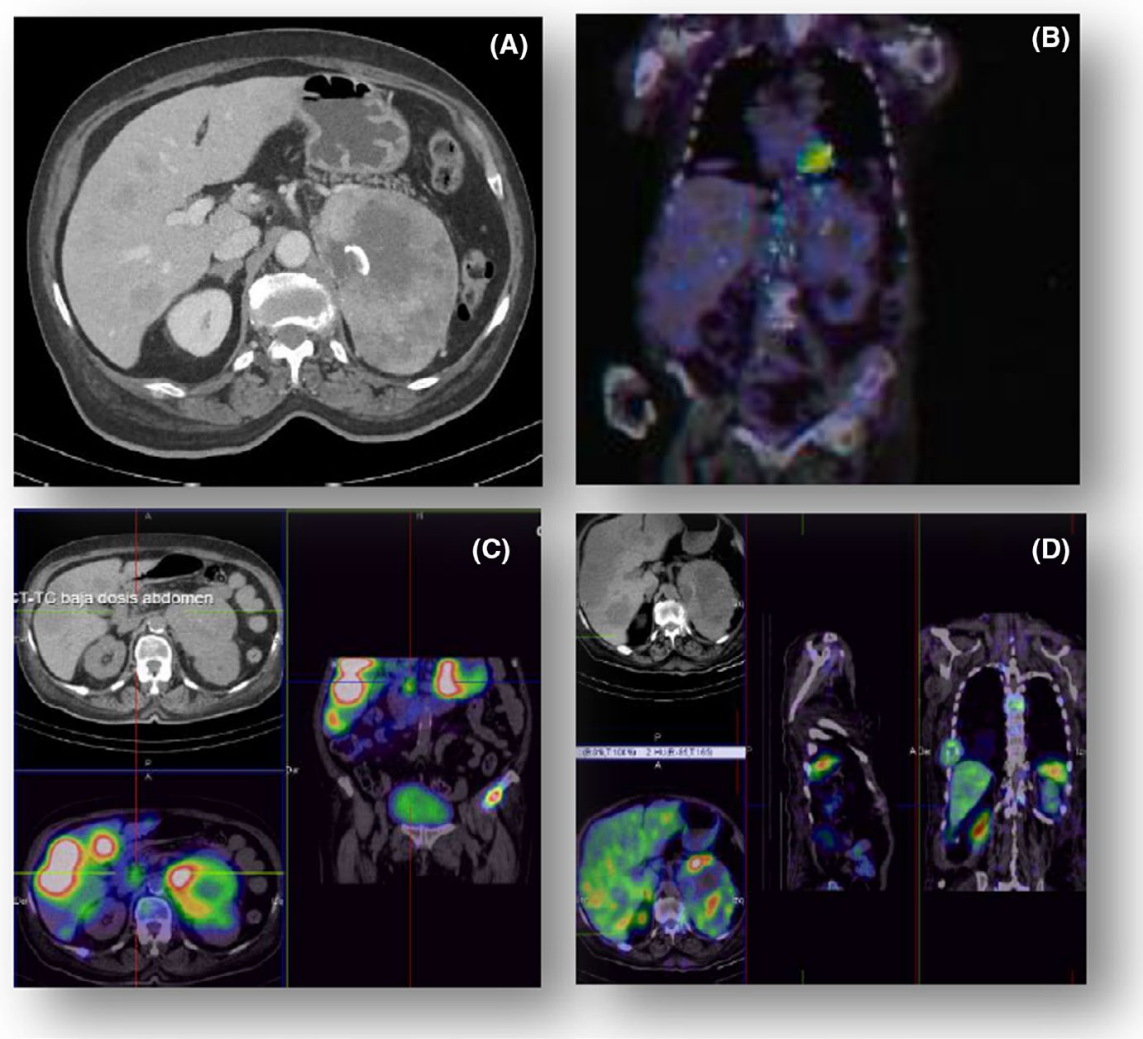
Contrast‐enhanced abdominal computed tomography (1A), F‐18 FDG PET/CT (1B), 123I‐MIBG scintigraphy (1C), and Technetium‐99m Octreotide scintigraphy (1D) of malignant pheochromocytoma

A sixty‐nine‐year‐old diabetic and hypertensive woman treated with insulin and oral hypoglycemic agents presented with a clinical course of costal and right hypochondrium pain, worsening of blood pressure measurements, and loss of weight for the last 2 months. A CT scan revealed a huge heterogeneous 91 × 90 × 137 mm adrenal left mass with irregular peripheral enhancement and suspicious lesions in bones originating possible medullar compression, lungs, liver, and a thoracic mass affecting soft tissues (Figure 1A). A hormonal profile was recommended which showed a huge increment in 24‐hour urinary metanephrines [normetanephrines: 3845 micrograms/24 hours (N: 162‐527), metanephrines: 3535,84 micrograms/24 hours (N:64‐302), and 3‐methoxytyramine: 947.81  micrograms/24 hours (N: 103‐434)], with high levels of neuron‐specific enolase (NSE): 2836 ng/mL (N < 15 ng/mL) and Chromogranin A: 680 000 ng/mL (N < 1019 ng/ml); the study was compatible with a malignant pheochromocytoma.

Surgery options were rejected due to high risk, illness spread, and bad prognosis. Selective alpha blockers (doxazosin) were prescribed with normalization of blood pressure, chemotherapy with cyclophosphamide, vincristine and dacarbazine started, and dorsal cementation and radiotherapy performed. Following two unsuccessful cycles of chemotherapy, radiological progression was observed and she presented with progressive hypoglycemia, requiring hospital admission due to lack of control at home.

After excluding adrenal and liver failure and sepsis, 2 days after hospitalization, a complete study of hypoglycemia showed the following results: glucose 32 mg/dL, insulin < 1 µU/mL, C‐peptide < 0.01 ng/mL and proinsulin < 0.6 pmol/L. Oral hypoglycemic agents and insulin antibodies were excluded. Furthermore, a hormonal evaluation included the following: GH 0.02 ng/mL, IGF1 < 15 ng/mL (N:42‐225 ng/mL) and IGF‐II 677 ng/mL (N:350‐1000 ng/mL). The IGF2/IGF1 ratio > 45(N < 10) guided to a NICTH diagnosis. However, it should be pointed that in sepsis or malnutrition, this ratio may increase (in these situations both IGFs are expected to be low) and conditions as renal failure may give a false‐negative ratio. A glucagon challenge was also performed, showing an increase of glucose levels from 32 mg/dL to 75 mg/dL at 15 minutes, suggesting a hormonal induced hypoglycemia. A PET scan showed a low glucose uptake in all lesions (max SUV3.2) (Figure 1B); therefore, hypoglycemia was not due to high glucose consumption by the tumor.

During several weeks, management of hypoglycemia required high‐dose corticosteroid therapy (12 mg/day of dexamethasone) besides enteral and parenteral nutrition with up to 500 grams of glucose. Due to a high tracer uptake, palliative treatment with MIBG (Figure 1C,D) was planned but was not administered because the patient quickly deteriorated and finally died 6 months after diagnosis.

## 2 DISCUSSION

Multiple actions of IGF‐II can be responsible for the development of hypoglycemia, most prominently by decreasing hepatic glucose output. GH, IGF‐1 and glucagon production are also suppressed by abnormal activation by IGF‐II of IGF‐1 receptors on the pancreatic alpha cells.

Under normal circumstances, IGF‐II binds with IGF‐binding protein‐3 to constitute a binary complex with hypoglycemic effects, unless it binds with an acid‐labile subunit to generate a ternary complex that confines inactive IGF‐II within the vascular space. A normal subject would present with just a 20% of IGF‐II in the binary complex. NICTH ratios of binary: ternary complexes are commonly reversed due to aberrant IGF‐II gene transcription and expression.^2^ Although high IGF‐II levels are typically reported, normal and even low levels have also been described^4^ and measurement of ternary forms of IGF‐II must be performed in a research laboratory setting because no commercially assay is currently available.

Tumoral surgical resection is the best treatment, frequently resulting in an immediate resolution of hypoglycemia. However, in many cases (including ours), total excision is either not feasible or delayed. Debulking should also be always pondered when complete surgical extirpation is not feasible. Radiation therapy, systemic targeted antitumor therapy, and other local modalities may be an option for some patients.

As a first step of treatment, an increase in oral frequency, amount, and caloric density of food intake (including oral glucose supplements) is often tried. When home management fails, treatment is usually focused on intravenous dextrose‐containing fluids (including parenteral nutrition) preventing further hypoglycemia in most cases. Admitting the limitations of continuous intravenous therapy, a rational alternative seldom employed may be continuous glucagon infusion. Glucocorticoids, though not always successful, can prevent hypoglycemia mainly by increasing hepatic gluconeogenesis^2^ but the dose needs to be constantly titrated (usually the equivalent of prednisone 30‐60 mg/day). Supraphysiological doses of 3‐12 mg/day of Recombinant human GH have otherwise been useful in some cases,^5^ but unlike steroid treatment, it fails to restore normal C‐peptide and insulin levels. Addition of the lowest possible doses to reduce glucocorticoid dose may yield a better therapeutic response, with a more bearable side effect profile than either agent by its own. Octreotide (even with tumor positivity on octreotide scintigraphy) and diazoxide (often limited by edema and fluid retention) have no role in NICTH.

## CONFLICT OF INTEREST

None declared.

## AUTHORS' CONTRIBUTION

MM: has directly contributed to the design and conception of the study and has obtained the biochemical results. In addition, she has elaborated the manuscript and searched the references. PT: has also contributed to the design of the study and provided the patient, collaborating in the manuscript edition, and reviewing the last edition. MEL, MG, AA, and ML: have all carefully reviewed the manuscript.

## ETHICAL APPROVAL

This paper was approved by the local hospital ethical committee.

## References

[1] Garla V , Sonani H , Palabindala V , Gómez‐Sánchez C , Subauste J , Lien LF . Non‐Islet cell hypoglycemia: case series and review of the literature. Front Endocrinol. 2019;10:316. 10.3389/fendo.2019.00316 PMC6529841 31156561

[2] Dynkevich Y , Rother KI , Whitford I , et al. Tumors, IGF‐2, and hypoglycemia: insights from the clinic, the laboratory, and the historical archive. Endocr Rev. 2013;34:798‐826. 23671155 10.1210/er.2012-1033

[3] Ronen JA , Gavin M , Ruppert MD , Peiris AN . Glycemic disturbances in pheochromocytoma and paraganglioma. Cureus. 2019;11(4):e4551. 31275775 10.7759/cureus.4551 PMC6592834

[4] Fukuda I , Hizuka N , Ishikawa Y , et al. Clinical features of insulin like growth factor‐II producing non‐islet‐cell tumor hypoglycemia. Growth Horm IGF Res. 2006;16:211‐216. 16860583 10.1016/j.ghir.2006.05.003

[5] Bodnar TW , Acevedo MJ , Pietropaolo M . Management of non‐islet‐cell tumor hypoglycemia: a clinical review. J Clin Endocrinol Metab. 2014;99(3):713‐722. 24423303 10.1210/jc.2013-3382 PMC5393479

